# An iterative algorithm for a system of generalized implicit variational inclusions

**DOI:** 10.1186/s40064-016-2916-8

**Published:** 2016-08-08

**Authors:** Iqbal Ahmad, Vishnu Narayan Mishra, Rais Ahmad, Mijanur Rahaman

**Affiliations:** 1 Department of Mathematics, Aligarh Muslim University, Aligarh, 202002 India; 2 Applied Mathematics and Humanties Department, Sardar Vallabhbhai National Institute of Technology, Ichchhanath Mahadev Dumas Road, Surat, 395 007 India

**Keywords:** Relaxed, Algorithm, Solution, Convergence, System, Resolvent, Primary 49J40, Secondary 90C33

## Abstract

In this paper, we introduce a system of generalized implicit variational inclusions which consists of three variational inclusions. We design an iterative algorithm with error terms based on relaxed resolvent operator due to Ahmad et al. (Stat Optim Inf Comput 4:183–193, 2016) for approximating the solution of our system. The convergence of the iterative sequences generated by the iterative algorithm is also discussed. An example is given which satisfy all the conditions of our main result.

## Background

A widely studied problem known as variational inclusion problem have many applications in the fields of optimization and control, economics and transportation equilibrium, engineering sciences, etc.. Several researches used different approaches to develop iterative algorithms for solving various classes of variational inequality and variational inclusion problems. For details see Ansari et al. (2000), Cho et al. (2004), Chang et al. (2005), Ding (2003), Fang and Huang (2004), Kim and Kim (2004), Kassay and Kolumbán (1999), Kassay et al. (2002), Kazmi et al. (2009), Lan et al. (2007), Noor (2001), Siddiqi et al. (1998), Sun et al. (2008), Yan et al. (2005) and the references therein.

A problem of much more interest called system of variational inequalities (inclusions) were introduced and studied in the literature. Peng (2003), Cohen and Chaplais (1988), Bianchi (1993), and Ansari and Yao (1999) considered a system of scalar variational inequalities and Pang showed that the traffic equilibrium problem, the spatial equilibrium problem, the Nash equilibrium, and the general equilibrium problem can be modeled as a system of variational inequalities. Verma (1999, 2001, 2004a, b) introduced and studied some systems of variational inequalities and developed some iterative algorithms for approximating the solutions of system of variational inequalities in Hilbert spaces.

As generalization of system of variational inequalities, Agarwal et al. (2004) introduced a system of generalized nonlinear mixed quasi-variational inclusions and studied the sensitivity analysis of solutions. After that, Fang and Huang (2004), Verma (2005), and Fang et al. (2005) introduced and studied different system of variational inclusions involving *H*-monotone operators, *A*-monotone operators, and $(H,\eta )$-monotone operators, respectively.

In this paper, we introduced and study a system of three variational inclusions and we call it system of generalized implicit variational inclusions in real Hilbert spaces. We design an iterative algorithm with error terms based on relaxed resolvent operator for solving system of generalized implicit variational inclusions. Convergence criteria is also discussed. The approach of this paper is different then the methods discussed above. An example is given in support of our main result.

## Preliminaries

Let *X* be a real Hilbert space endowed with a norm $$\Vert \cdot \Vert$$ and an inner product $$\langle \cdot ,\cdot \rangle ,$$*d* is the metric induced by the norm $$\Vert \cdot \Vert ,$$$$2^{X}$$ (respectively,*CB*(*X*)) is the family of all nonempty (respectively, closed and bounded) subsets of *X*,  and $$D(\cdot ,\cdot )$$ is the Hausdörff metric on *CB*(*X*) defined by

$$\begin{aligned} D(P,Q)=\max \left\{ \sup \limits _{x\in P}d(x,Q), \sup \limits _{y\in Q}d(P,y)\right\} , \end{aligned}$$

where $$d(x,Q)=\inf \nolimits _{y\in Q}d(x,y)$$ and $$d(P,y)=\inf \nolimits _{x\in P}d(x,y)$$.

Let us recall the known definitions needed in the sequel.

### **Definition 1**

A mapping $g: X \rightarrow X$ is said to be

- (*i*) Lipschitz continuous if, there exists a constant $\lambda _{g}>0$ such that
  $$\begin{aligned} \Vert g(x)-g(y)\Vert \le \lambda _{g}\Vert x-y\Vert , \quad \forall x,y\in X; \end{aligned}$$
- (*ii*) monotone if,
  $$\begin{aligned} \langle g(x)-g(y),x-y\rangle \ge 0, \quad \forall x,y\in X; \end{aligned}$$
- (*iii*) strongly monotone if, there exists a constant $\xi >0$ such that
  $$\begin{aligned} \langle g(x)-g(y),x-y\rangle \ge \xi \Vert x-y\Vert ^2, \quad \forall x,y\in X; \end{aligned}$$
- (*iv*) relaxed Lipschitz continuous if, there exists a constant $r>0$ such that
  $$\begin{aligned} \langle g(x)-g(y),x-y\rangle \le -r\Vert x-y\Vert ^2, \quad \forall x,y\in X. \end{aligned}$$

### **Definition 2**

A mapping $F:X\times X\times X \rightarrow X$ is said to be Lipschitz continuous with respect to first argument if, there exists a constant $\lambda _{F_1}$ such that

$$\begin{aligned} \Vert F(x_1,x_2,x_3)-F(y_1,x_2,x_3)\Vert \le \lambda _{F_1} \Vert x_1-y_1\Vert , \quad \forall x_1, y_1, x_2,x_3\in X. \end{aligned}$$

Similarly, we can define the Lipschitz continuity of *F* in rest of the arguments.

### **Definition 3**

A set-valued mapping $A:X\rightarrow CB(X)$ is said to be *D*-Lipschitz continuous if, there exists a constant $\delta _{A}$ such that

$$\begin{aligned} D(A(x),A(y))\le \delta _{A} \Vert x-y\Vert , \quad \forall x, y\in X. \end{aligned}$$

### **Definition 4**

Ahmad et al. (2016) Let $H:X\rightarrow X$ be a mapping and $I:X\rightarrow X$ be an identity mapping. Then, a set-valued mapping $M:X\rightarrow 2^X$ is a said to be $(I-H)$-monotone if, *M* is monotone, *H* is relaxed Lipschitz continuous and

$$\begin{aligned}{}[(I-H)+\lambda M](X)=X, \end{aligned}$$

where $\lambda >0$ is a constant.

### **Definition 5**

Ahmad et al. (2016) Let $H:X\rightarrow X$ be relaxed Lipschitz continuous mapping and $I:X\rightarrow X$ be an identity mapping. Suppose that $M:X\rightarrow 2^X$ is a set-valued, $(I-H)$-monotone mapping. The relaxed resolvent operator $R^{(I-H)}_{\lambda ,M}:X\rightarrow X$ associated with *I*,*H* and *M* is defined by

1 $$\begin{aligned} R^{I-H}_{\lambda ,M}(x)=[(I-H)+\lambda M]^{-1}(x), \quad \forall x\in X, \end{aligned}$$

where $\lambda >0$ is a constant.

For the sake of convenience of readers, we give the proof following two theorems which can be found in Ahmad et al. (2016).

### **Theorem 1**

*Let*$H:X\rightarrow X$*be anr-relaxed Lipschitz continuous mapping,*$I:X\rightarrow X$*be an identity mapping and*$M:X\rightarrow 2^X$*be a set-valued*, $(I-H)$*-monotone mapping. Then the operator*$[(I-H)+\lambda M]^{-1}$*is single-valued, where*$\lambda >0$*is a constant*.

### *Proof*

For any $z\in X$ and a constant $\lambda >0,$ let $x,y\in [(I-H)+\lambda M]^{-1}(z).$ Then,

$$\begin{aligned}&\lambda ^{-1}[z-(I-H)(x)]\in M(x);\\&\lambda ^{-1}[z-(I-H)(y)]\in M(y). \end{aligned}$$

Since *M* is monotone, we have

$$\begin{aligned} \langle -(I-H)(x)+z+(I-H)(y)-z,x-y\rangle &\ge {} 0;\\ -\langle (I-H)(x)-(I-H)(y),x-y\rangle & \ge {} 0;\\ -\langle x-H(x)-y+H(y),x-y\rangle & \ge {} 0;\\ \langle x-H(x)-y+H(y),x-y\rangle &\le {} 0;\\ \langle x-H(x)-y+H(y),x-y\rangle & \le {} 0;\\ \langle x-y,x-y\rangle -\langle H(x)-H(y),x-y\rangle & \le {} 0. \end{aligned}$$

Since *H* is *r*-relaxed Lipschitz continuous, we have

$$\begin{aligned} 0\ge \langle x-y,x-y\rangle -\langle H(x)-H(y),x-y\rangle \ge \Vert x-y\Vert ^{2}+r\Vert x-y\Vert ^{2} \ge 0, \end{aligned}$$

it follows that $(1+r)\Vert x-y\Vert ^{2}=0,$ which implies that $x=y.$ Thus $[(I-H)+\lambda M]^{-1}$ is single-valued. $\square$

### **Theorem 2**

*Let*$H:X\rightarrow X$*be anr-relaxed Lipschitz continuous mapping*, $I:X\rightarrow X$*be an identity mapping and*$M:X\rightarrow 2^{X}$*be a set-valued*, $(I-H)$*-monotone mapping. Then the relaxed resolvent operator*$R^{I-H}_{\lambda ,M}:X\rightarrow X$*is*$\frac{1}{1+r}$*-Lipschitz continuous. i.e.*,

$$\begin{aligned} \Vert R^{I-H}_{\lambda ,M}(x)-R^{I-H}_{\lambda ,M}(y)\Vert \le \frac{1}{1+r}\Vert x-y\Vert , \quad \forall x,y\in X. \end{aligned}$$

### *Proof*

Let *x* and *y* be any given point in *X*. If follow from (1) that

2 $$\begin{aligned} \begin{aligned} R^{I-H}_{\lambda ,M}(x)=[(I-H)+\lambda M]^{-1}(x),\\ R^{I-H}_{\lambda ,M}(y)=[(I-H)+\lambda M]^{-1}(y), \end{aligned} \end{aligned}$$

i.e.,

3 $$\begin{aligned} \begin{aligned} \frac{1}{\lambda }\left[ x-(I-H)(R^{I-H}_{\lambda ,M}(x))\right] \in M \left( R^{I-H}_{\lambda ,M}(x)\right) ,\\ \frac{1}{\lambda }\left[ y-(I-H)(R^{I-H}_{\lambda ,M}(y))\right] \in M \left( R^{I-H}_{\lambda ,M}(y)\right) . \end{aligned} \end{aligned}$$

Since *M* is $(I-H)$-monotone i.e., *M* is monotone, we have

4 $$\begin{aligned} \begin{aligned} \frac{1}{\lambda }\left\langle x-(I-H)(R^{I-H}_{\lambda ,M}(x))-(y-(I-H)(R^{I-H}_{\lambda ,M}(y))),R^{I-H}_{\lambda ,M}(x)-R^{I-H}_{\lambda ,M}(y)\right\rangle \ge 0,\\ \frac{1}{\lambda }\left\langle x-y-\{(I-H)(R^{I-H}_{\lambda ,M}(x))-(I-H)(R^{I-H}_{\lambda ,M}(y))\},R^{I-H}_{\lambda ,M}(x)-R^{I-H}_{\lambda ,M}(y)\right\rangle \ge 0. \end{aligned} \end{aligned}$$

It follows that

5 $$\begin{aligned}&\left\langle x-y,R^{I-H}_{\lambda ,M}(x)-R^{I-H}_{\lambda ,M}(y)\right\rangle \nonumber \\&\quad \ge \left\langle (I-H)(R^{I-H}_{\lambda ,M}(x))-(I-H)(R^{I-H}_{\lambda ,M}(y)),R^{I-H}_{\lambda ,M}(x)-R^{I-H}_{\lambda ,M}(y)\right\rangle . \end{aligned}$$

By Cauchy-Schwartz inequality, (5) and *r*-relaxed Lipschitz continuity of *H*,  we have

6 $$\begin{aligned}&\Big \Vert x-y\Big \Vert \left\| R^{I-H}_{\lambda ,M}(x)-R^{I-H}_{\lambda ,M}(y)\right\| \nonumber \\&\quad \ge \left\langle x-y,R^{I-H}_{\lambda ,M}(x)-R^{I-H}_{\lambda ,M}(y)\right\rangle \nonumber \\&\quad \ge \left\langle R^{I-H}_{\lambda ,M}(x)-H(R^{I-H}_{\lambda ,M}(x))-R^{I-H}_{\lambda ,M}(y)+H(R^{I-H}_{\lambda ,M}(y)),R^{I-H}_{\lambda ,M}(x)-R^{I-H}_{\lambda ,M}(y)\right\rangle \nonumber \\&\quad =\left\langle R^{I-H}_{\lambda ,M}(x)-R^{I-H}_{\lambda ,M}(y),R^{I-H}_{\lambda ,M}(x)-R^{I-H}_{\lambda ,M}(y)\right\rangle -\nonumber \\&\quad \left\langle H(R^{I-H}_{\lambda ,M}(x))-H(R^{I-H}_{\lambda ,M}(y)),R^{I-H}_{\lambda ,M}(x)-R^{I-H}_{\lambda ,M}(y)\right\rangle \nonumber \\&\quad \ge \left\| R^{I-H}_{\lambda ,M}(x)-R^{I-H}_{\lambda ,M}(y)\right\| ^2+r\left\| R^{I-H}_{\lambda ,M}(x)-R^{I-H}_{\lambda ,M}(y)\right\| ^2\nonumber \\&\quad =(1+r)\left\| R^{I-H}_{\lambda ,M}(x)-R^{I-H}_{\lambda ,M}(y)\right\| ^2. \end{aligned}$$

Thus, we have

$$\begin{aligned} \left\| R^{I-H}_{\lambda ,M}(x)-R^{I-H}_{\lambda ,M}(y)\right\| \le \frac{1}{1+r}\Vert x-y\Vert , \end{aligned}$$

i.e., the relaxed resolvent operator $R^{I-H}_{\lambda ,M}$ is $\frac{1}{1+r}$-Lipschitz continuous. $\square$

## System of generalized implicit variational inclusions and iterative algorithm

In this section, we introduce a system of generalized implicit variational inclusions and design an iterative algorithm with error terms for solving the system of generalized implicit variational inclusions in Hilbert spaces.

For each $$i\in \{1,2,3\},$$ let $$X_i$$ be a real Hilbert space, $$H_i,g_i:X_i\rightarrow X_i,$$$$F_i,P_i:X_1\times X_2\times X_3\rightarrow X_i$$ be the single-valued mappings and $$A_{i1},A_{i2},A_{i3}:X_i\rightarrow CB(X_i)$$ be the set-valued mappings. Let $$I_i:X_i\rightarrow X_i$$ be the identity mappings and $$M_i:X_i\times X_i\rightarrow 2^{X_i}$$ be the set-valued, $$(I_i-H_i)$$-monotone mappings. We consider the following system of generalized implicit variational inclusions (in short, SGIVI):

Find $$(x_1,x_2,x_3,u_{11},u_{12},u_{13},u_{21},u_{22},u_{23},u_{31},u_{32},u_{33})$$ such that for each $$i\in \{1,2,3\},$$$$(x_1,x_2,x_3)\in X_1\times X_2\times X_3,$$$$u_{i1}\in A_{i1}(x_1),$$$$u_{i2}\in A_{i2}(x_2),$$$$u_{i3}\in A_{i3}(x_3)$$ such that

7 $$\begin{aligned} {\left\{ \begin{array}{ll} 0\in F_1(x_1,x_2,x_3)+P_1(u_{11},u_{12},u_{13})+M_1(g_1(x_1),x_1),\\ 0\in F_2(x_1,x_2,x_3)+P_2(u_{21},u_{22},u_{23})+M_2(g_2(x_2),x_2),\\ 0\in F_3(x_1,x_2,x_3)+P_3(u_{31},u_{32},u_{33})+M_3(g_3(x_3),x_3). \end{array}\right. } \end{aligned}$$

Let us see some special cases of SGIVI (7) below.

- (*i*) If $$F_1(x_1,x_2,x_3)\equiv F(x_1,x_2),$$$$F_2(x_1,x_2,x_3)\equiv G(x_1,x_2),$$$$F_3\equiv 0,$$$$P_1(.,.,.)\equiv P(.,.),$$$$P_2(.,.,.)\equiv Q(.,.),$$$$P_3\equiv 0,$$$$M_1(g_1(x_1),x_1)\equiv M_1(g_1(x_1)),$$$$M_2(g_2(x_2),x_2)\equiv M_2(g_2(x_2)),$$$$M_3\equiv 0,$$ then problem (7) reduces to the system of generalized mixed quasi-variational inclusions with $$(H,\eta )$$-monotone operators, which is to find $$(x_1,x_2)\in X_1\times X_2$$ such that
  8 $$\begin{aligned} {\left\{ \begin{array}{ll} 0\in F(x_1,x_2)+P(u,v)+M_1(g_1(x_1)),\\ 0\in G(x_1,x_2)+Q(w,z)+M_2(g_2(x_2)). \end{array}\right. } \end{aligned}$$
  Problem (8) was introduced and studied by Peng and Zhu (2007).
- (*ii*) If $$F_1(x_1,x_2,x_3)\equiv F(x_1,x_2),$$$$F_2(x_1,x_2,x_3)\equiv G(x_1,x_2),$$$$F_3\equiv 0,$$$$P_1=P_2=P_3\equiv 0,$$$$g_1\equiv I_1$$(the identity map on $$X_1$$), $$g_2\equiv I_2$$ (the identity map on $$X_2$$) $$g_3\equiv 0,$$$$M_1(g_1(x_1),x_1)\equiv M_1(x_1),$$$$M_2(g_2(x_2),x_2)\equiv M_2(x_2),$$$$M_3\equiv 0,$$ then problem (7) reduces to the system of variational inclusions with $$(H,\eta )$$-monotone operators, which is to find $$(x,y)\in X_1\times X_2$$ such that
  9 $$\begin{aligned} {\left\{ \begin{array}{ll} 0\in F(x_1,x_2)+M_1(x_1),\\ 0\in G(x_1,x_2)+M_2(x_2). \end{array}\right. } \end{aligned}$$
  Problem (9) was introduced and studied by Fang et al. (2005).

Now, we mention the following fixed point formulation of SGIVI (7).

### **Lemma 1**

*For each*$$i\in \{1,2,3\},$$*let*$$X_i$$*be a real Hilbert space*, $$H_i,g_i:X_i\rightarrow X_i,$$$$F_i,P_i:X_1\times X_2\times X_3\rightarrow X_i$$*be single-valued mappings and*$$A_{i1},A_{i2},A _{i3}:X_i\rightarrow CB(X_i)$$*be the set-valued mappings. Let*$$I_i:X_i\rightarrow X_i$$*be the identity mappings and*$$M_i:X_i\times X_i\rightarrow 2^{X_i}$$*be the set-valued*, $$(I_i-H_i)$$*-monotone mappings. Then*$$(x_1,x_2,x_3,u_{11},u_{12},u_{13},u_{21},u_{22},u_{23},u_{31},u_{32},u_{33})$$*with*$$(x_1,x_2,x_3)\in X_1\times X_2\times X_3,$$$$u_{i1}\in A_{i1}(x_1),$$$$u_{i2}\in A_{i2}(x_2),$$$$u_{i3}\in A_{i3}(x_3)$$*is a solution of SGIVI* (7), *if and only if the following equations are satisfied:*

$$\begin{aligned} g_i(x_i)&= {} R^{I_i-H_i}_{\lambda _i,M_i(.,x_i)}[(I_i-H_i)(g_i(x_i))-\lambda _iF_i(x_1,x_2,x_3)-\lambda _iP_i(u_{i1},u_{i2},u_{i3})], \end{aligned}$$

*where*$R^{I_i-H_i}_{\lambda _i,M_i(.,x_i)}=[(I_i-H_i)+\lambda _iM_i(.,x_i)]^{-1}$*are the relaxed resolvent operators and*$\lambda _i>0$*are constants*.

### *Proof*

The proof is a direct consequence of the definition of the relaxed resolvent operator. $\square$

We design the following iterative algorithm with error terms to approximate the solution of SGIVI (7).

### **Iterative Algorithm 1**

For each $$i\in \{1,2,3\},$$ given $$x^{0}_{i}\in X_i,$$ take $$u^{0}_{i1}\in A_{i1}(x^{0}_{1}),$$$$u^{0}_{i2}\in A_{i2}(x^{0}_{2}),$$$$u^{0}_{i3}\in A_{i3}(x^{0}_{3})$$ and let

$$\begin{aligned} x^{1}_{i} &= {} (1-\mu _i)x^{0}_{i}+\mu _i[x^{0}_{i}-g_i(x^{0}_{i})+R^{I_i-H_i}_{\lambda _i,M_i(.,x^{0}_i)}((I_i-H_i)(g_i(x^{0}_i))-\lambda _iF_i(x^{0}_{1},x^{0}_{2},x^{0}_{3})\\&\quad-\,\lambda _iP_i(u^{0}_{i1},u^{0}_{i2},u^{0}_{i3}))]+\mu _ie^{0}_{i}. \end{aligned}$$

*Since*$$u^{0}_{i1}\in A_{i1}(x^{0}_{1}),$$$$u^{0}_{i2}\in A_{i2}(x^{0}_{2}),$$$$u^{0}_{i3}\in A_{i3}(x^{0}_{3}),$$ by Nadler’s (1992) theorem, *there exist*$$u^{1}_{i1}\in A_{i1}(x^{1}_{1}),$$$$u^{1}_{i2}\in A_{i2}(x^{1}_{2}),$$$$u^{1}_{i3}\in A_{i3}(x^{1}_{3}),$$ such that

$$\begin{aligned} \Vert u^{1}_{i1}-u^{0}_{i1}\Vert &\le {} (1+1)D_1(A_{i1}(x^{1}_1),A_{i1}(x^{0}_{1}),\\ \Vert u^{1}_{i2}-u^{0}_{i2}\Vert &\le {} (1+1)D_2(A_{i2}(x^{1}_{2}),A_{i2}(x^{0}_{2})),\\ \Vert u^{1}_{i3}-u^{0}_{i3}\Vert & \le {} (1+1)D_3(A_{i3}(x^{1}_{3}),A_{i3}(x^{0}_{3})). \end{aligned}$$

Again, let

$$\begin{aligned} x^{2}_{i}&= {} (1-\mu _i)x^{1}_{i}+\mu _i[x^{1}_{i}-g_i(x^{1}_{i})+R^{I_i-H_i}_{\lambda _i,M_i(.,x^{1}_i)}((I_i-H_i)(g_i(x^{1}_i))-\lambda _iF_i(x^{1}_{1},x^{1}_{2},x^{1}_{3})\\&\;-\,\lambda _iP_i(u^{1}_{i1},u^{1}_{i2},u^{1}_{i3}))]+\mu _ie^{1}_{i}. \end{aligned}$$

By Nadler’s (1992) theorem, there exist $$u^{2}_{i1}\in A_{i1}(x^{2}_{1}),$$$$u^{2}_{i2}\in A_{i2}(x^{2}_{2}),$$$$u^{2}_{i3}\in A_{i3}(x^{2}_{3})$$ such that

$$\begin{aligned} \Vert u^{2}_{i1}-u^{1}_{i1}\Vert & \le {} \left( 1+\frac{1}{2}\right) D_1(A_{i1}(x^{2}_1),A_{i1}(x^{1}_{1}),\\ \Vert u^{2}_{i2}-u^{1}_{i2}\Vert & \le {} \left( 1+\frac{1}{2}\right) D_2(A_{i2}(x^{2}_{2}),A_{i2}(x^{1}_{2})),\\ \Vert u^{2}_{i3}-u^{1}_{i3}\Vert & \le {} \left( 1+\frac{1}{2}\right) D_3(A_{i3}(x^{2}_{3}),A_{i3}(x^{1}_{3})). \end{aligned}$$

Continuing the above process inductively, we can obtain the sequences $$\{x^{n}_{i}\},$$$$\{u^{n}_{i1}\},$$$$\{u^{n}_{i2}\},$$$$\{u^{n}_{i3}\}$$ by the following iterative schemes:

10 $$\begin{aligned} x^{n+1}_{i}&= {} (1-\mu _i)x^{n}_{i}+\mu _i[x^{n}_{i}-g_i(x^{n}_{i})+R^{I_i-H_i}_{\lambda _i,M_i(.,x^{n}_i)}((I_i-H_i)(g_i(x^{n}_i))\nonumber \\&\quad -\lambda _iF_i(x^{n}_{1},x^{n}_{2},x^{n}_{3})-\lambda _iP_i(u^{n}_{i1},u^{n}_{i2},u^{n}_{i3}))]+\mu _ie^{n}_{i}. \end{aligned}$$

11 $$\begin{aligned} \Vert u^{n+1}_{i1}-u^{n}_{i1}\Vert &\le {} \left( 1+\frac{1}{n+1}\right) D_1(A_{i1}(x^{n+1}_1),A_{i1}(x^{n}_{1}), \end{aligned}$$

12 $$\begin{aligned} \Vert u^{n+1}_{i2}-u^{n}_{i2}\Vert &\le {} \left( 1+\frac{1}{n+1}\right) D_2(A_{i2}(x^{n+1}_{2}),A_{i2}(x^{n}_{2})), \end{aligned}$$

13 $$\begin{aligned} \Vert u^{n+1}_{i3}-u^{n}_{i3}\Vert &\le {} \left( 1+\frac{1}{n+1}\right) D_3(A_{i3}(x^{n+1}_{3}),A_{i3}(x^{n}_{3})), \end{aligned}$$

where $$n=0,1,2\ldots ,$$ for $$i\in \{1,2,3\},$$$$\mu _i>0,$$$$\lambda _i>0$$ are constants, $$e^{n}_{i}\in X_i \,(n\ge 0)$$ are errors to take into account a possible inexact computation of the resolvent operator point and $$D_i(.,.)$$ are the Hausdorff metrics on $$CB(X_i).$$

## An existence and convergence result

In this section, we will prove an existence result for SGIVI (7) and we show the convergence of iterative sequences generated by Algorithm 1, which is our main motive.

### **Theorem 3**

*For each*$$i\in \{1,2,3\},$$*let*$$X_i$$*be a Hilbert space*, $$I_i:X_i\rightarrow X_i$$*be the identity mappings and*$$H_i,g_i:X_i\rightarrow X_i$$*be the single-valued mappings such that*$$g_i$$*is*$$\xi _i$$*-strongly monotone*, $$\lambda _{g_i}$$*-Lipschitz continuous and*$$H_i$$*is*$$\lambda _{H_i}$$*-Lipschitz continuous*, $$r_i$$*-relaxed Lipschitz continuous. Suppose that*$$A_{i1},A_{i2},A_{i3}:X_i\rightarrow CB(X_i)$$*are the set-valued mappings such that*$$A_{i1}$$*is*$$\delta _{A_{i1}}$$-$$D_{1}$$*-Lipschitz continuous*, $$A_{i2}$$*is*$$\delta _{A_{i2}}$$-$$D_{2}$$*-Lipschitz continuous and*$$A_{i3}$$*is*$$\delta _{A_{i3}}$$-$$D_{3}$$-*Lipschitz continuous, respectively. Let*$$F_i,P_i:X_1\times X_2\times X_3\rightarrow X_i$$*be the single-valued mappings such that*$$F_i$$’*sare Lipschitz continuous in all three arguments with constants*$$\lambda _{F_{i1}}>0,$$$$\lambda _{F_{i2}}>0,$$$$\lambda _{F_{i3}}>0,$$*respectively and*$$P_i$$’*s are Lipschitz continuous in all three arguments with constants*$$\lambda _{P_{i1}}>0,$$$$\lambda _{P_{i2}}>0,$$$$\lambda _{P_{i3}}>0,$$*respectively. Suppose that*$$M_i:X_i\times X_i\rightarrow 2^{X_i}$$*are the set-valued*, $$(I_i-H_i)$$*-monotone mappings. Assume that there exist constants*$$\lambda _i>0$$*and*$$h_i>0$$*such that the following conditions hold:*

14 $$\begin{aligned} \left\| R^{I_i-H_i}_{\lambda _i,M_i(.,x)}(z)-R^{I_i-H_i}_{\lambda _i,M_i(.,y)}(z)\right\| \le h_i\Vert x-y\Vert ,\quad \forall x,y,z\in X_i, \end{aligned}$$

*and*

15 $$\begin{aligned} {\left\{ \begin{array}{ll} \kappa _i=1-\mu _i+\mu _ih_i+\mu _i\sqrt{1-2\xi _i+\lambda ^{2}_{g_i}}+\frac{\mu _i\lambda _{g_i}+\mu _i\lambda _{H_i}\lambda _{g_i}}{1+r_i}+\sum \limits ^{3}_{j=1}\frac{\mu _j\lambda _j\lambda _{F_{ji}}}{1+r_j}<1,\\ \nu _i=\mu _i\left( \sum \limits ^{3}_{j=1}\frac{\mu _j\lambda _j\lambda _{P_{ji}}\delta _{A_{ji}}}{1+r_j}\right)<1,\\ \kappa _i+\nu _i<1\,and\,2\xi _i<1+\lambda ^{2}_{g_i},\quad for\,each\,i\in \{1,2,3\},\\ \sum \limits ^{\infty }_{q=1}\Vert e^{q}_{1}-e^{q-1}_{1}\Vert \kappa ^{-q}<\infty , \sum \limits ^{\infty }_{q=1}\Vert e^{q}_{2}-e^{q-1}_{2}\Vert \kappa ^{-q}<\infty ,\\ \sum \limits ^{\infty }_{q=1}\Vert e^{q}_{3}-e^{q-1}_{3}\Vert \kappa ^{-q}<\infty ,\\ \lim \limits _{n\rightarrow \infty }e^{n}_{1}=\lim \limits _{n\rightarrow \infty }e^{n}_{2}=\lim \limits _{n\rightarrow \infty }e^{n}_{3}=0,\quad for\,each\,\kappa \in (0,1). \end{array}\right. } \end{aligned}$$

*Then, the SGIVI* (7) *admits a solution*$$(x_1,x_2,x_3,u_{11},u_{12},u_{13},u_{21},u_{22},u_{23},u_{31},u_{32},u_{33})$$*and the iterative sequences*$$\{x^{n}_{i}\},$$$$\{u^{n}_{i1}\},$$$$\{u^{n}_{i2}\},$$$$\{u^{n}_{i3}\}$$*generated by iterative Algorithm*1*strongly converge to*$$x_i,$$$$u_{i1},$$$$u_{i2},$$$$u_{i3},$$*respectively, for each*$$i\in \{1,2,3\}.$$

### *Proof*

For each $i\in \{1,2,3\},$ let $d^{n}_i=[(I_i-H_i)(g_i(x^{n}_{,i}))-\lambda _iF_i(x^{n}_1,x^{n}_2,x^{n}_3)- \lambda _iP_i(u^{n}_{i1},u^{n}_{i2},u^{n}_{i3})].$

Using Algorithm 1, condition (14) and Theorem 2, we have

16 $$\begin{aligned}&\Vert x^{n+1}_{1}-x^n_{1}\Vert \nonumber \\&\quad =\Vert (1-\mu _1)x^{n}_{1}+\mu _1[x^{n}_{1}-g_1(x^{n}_{1})+R^{I_1-H_1}_{\lambda _1,M_1(.,x^{n}_1)}(d^{n}_{1})]+\mu _1e^{n}_{1}-(1-\mu _1)x^{n-1}_{1}\nonumber \\&\quad -\mu _1[x^{n-1}_{1}-g_1(x^{n-1}_{1})+R^{I_1-H_1}_{\lambda _1,M_1(.,x^{n-1}_1)}(d^{n-1}_{1})]-\mu _1e^{n-1}_{1}\Vert \nonumber \\&\quad \le (1-\mu _1)\Vert x^{n}_{1}-x^{n-1}_{1}\Vert +\mu _1\Vert x^{n}_{1}-x^{n-1}_{1}-(g_1(x^{n}_{1})-g_1(x^{n-1}_{1}))\Vert \nonumber \\&\quad +\mu _1\Vert R^{I_1-H_1}_{\lambda _1,M_1(.,x^{n}_1)}(d^{n}_{1})-R^{I_1-H_1}_{\lambda _1,M_1(.,x^{n}_1)}(d^{n-1}_{1})\Vert +\mu _1\Vert R^{I_1-H_1}_{\lambda _1,M_1(.,x^{n}_1)}(d^{n-1}_{1})\nonumber \\&\quad -R^{I_1-H_1}_{\lambda _1,M_1(.,x^{n-1}_1)}(d^{n-1}_{1})\Vert +\mu _1\Vert e^{n}_{1}-e^{n-1}_{1}\Vert \nonumber \\&\quad \le (1-\mu _1)\Vert x^{n}_{1}-x^{n-1}_{1}\Vert +\mu _1\Vert x^{n}_{1}-x^{n-1}_{1}-(g_1(x^{n}_{1})-g_1(x^{n-1}_{1}))\Vert \nonumber \\&\quad +\frac{\mu _1}{1+r_1}\Vert d^{n}_{1}-d^{n-1}_{1}\Vert +\mu _1h_1\Vert x^{n}_{1}-x^{n-1}_{1}\Vert +\mu _1\Vert e^{n}_{1}-e^{n-1}_{1}\Vert \nonumber \\&\quad \le (1-\mu _1+\mu _1h_1)\Vert x^{n}_{1}-x^{n-1}_{1}\Vert +\mu _1\Vert x^{n}_{1}-x^{n-1}_{1}-(g_1(x^{n}_{1})-g_1(x^{n-1}_{1}))\Vert \nonumber \\&\quad +\frac{\mu _1}{1+r_1}\Vert d^{n}_{1}-d^{n-1}_{1}\Vert +\mu _1\Vert e^{n}_{1}-e^{n-1}_{1}\Vert . \end{aligned}$$

As $g_1$ is $\xi _1$-strongly monotone and $\lambda _{g_1}$-Lipschitz continuous, we obtain

17 $$\begin{aligned}&\Vert x^{n}_{1}-x^{n-1}_{1}-(g_1(x^{n}_{1})-g_1(x^{n-1}_{1}))\Vert ^2\nonumber \\&\quad =\Vert x^{n}_{1}-x^{n-1}_{1}\Vert ^2-2\left\langle x^{n}_{1}-x^{n-1}_{1},g_1(x^{n}_{1})-g_1(x^{n-1}_{1})\right\rangle +\Vert g_1(x^{n}_{1})-g_1(x^{n-1}_{1})\Vert ^2\nonumber \\&\quad \le (1-2\xi _1+\lambda ^{2}_{g_1})\Vert x^{n}_{1}-x^{n-1}_{1}\Vert ^2. \end{aligned}$$

As $$g_1$$ is $$\lambda _{g_1}$$-Lipschitz continuous, $$F_1$$ is Lipschitz continuous in all three arguments with constants $$\lambda _{F_{11}},$$$$\lambda _{F_{12}}$$ and $$\lambda _{F_{13}},$$ respectively, $$P_1$$ is Lipschitz continuous in all three arguments with constants $$\lambda _{P_{11}},$$$$\lambda _{P_{12}}$$ and $$\lambda _{P_{13}},$$ respectively, $$A_{11}$$ is $$\delta _{A_{11}}$$-$$D_1$$-Lipschitz continuous, $$A_{12}$$ is $$\delta _{A_{12}}$$-$$D_2$$-Lipschitz continuous and $$A_{13}$$ is $$\delta _{A_{13}}$$-$$D_3$$-Lipschitz continuous, respectively, we obtain

18 $$\begin{aligned}&\Vert d^{n}_{1}-d^{n-1}_{1}\Vert \nonumber \\&\quad =\Vert (I_1-H_1)(g_1(x^{n}_{1}))-\lambda _1F_1(x^{n}_{1},x^{n}_{2},x^{n}_{3})-\lambda _1P_1(u^{n}_{11},u^{n}_{12},u^{n}_{13})\nonumber \\&\quad -(I_1-H_1)(g_1(x^{n-1}_{1}))+\lambda _1F_1(x^{n-1}_{1},x^{n-1}_{2},x^{n-1}_{3})+\lambda _1P_1(u^{n-1}_{11},u^{n-1}_{12},u^{n-1}_{13})\Vert \nonumber \\&\quad \le \Vert g_1(x^{n}_{1})-g_1(x^{n-1}_{1})\Vert +\Vert H_1(g_1(x^{n}_1))-H_1(g_1(x^{n-1}_1))\Vert \nonumber \\&\quad +\lambda _1\Vert F_1(x^{n}_1,x^{n}_2,x^{n}_3)-F_1(x^{n-1}_1,x^{n-1}_2,x^{n-1}_3)\Vert +\lambda _1\Vert P_1(u^{n}_{11},u^{n}_{12},u^{n}_{13})\nonumber \\&\quad -P_1(u^{n-1}_{11},u^{n-1}_{12},u^{n-1}_{13})\Vert \nonumber \\&\quad \le \Vert g_1(x^{n}_1)-g_1(x^{n-1}_1)\Vert +\Vert H_1(g_1(x^{n}_1))-H_1(g_1(x^{n-1}_1))\Vert +\lambda _1\Vert F_1(x^{n}_1,x^{n}_2,x^{n}_3)\nonumber \\&\quad -F_1(x^{n-1}_1,x^{n}_2,x^{n}_3)\Vert +\lambda _1\Vert F_1(x^{n-1}_1,x^{n}_2,x^{n}_3)-F_1(x^{n-1}_1,x^{n-1}_2,x^{n}_3)\Vert \nonumber \\&\quad +\lambda _1\Vert F_1(x^{n-1}_1,x^{n-1}_2,x^{n}_3)-F_1(x^{n-1}_1,x^{n-1}_2,x^{n-1}_3)\Vert +\lambda _1\Vert P_1(u^{n}_{11},u^{n}_{12},u^{n}_{13})\nonumber \\&\quad -P_1(u^{n-1}_{11},u^{n}_{12},u^{n}_{13})\Vert +\lambda _1\Vert P_1(u^{n-1}_{11},u^{n}_{12},u^{n}_{13})-P_1(u^{n-1}_{11},u^{n-1}_{12},u^{n}_{13})\Vert \nonumber \\&\quad +\lambda _1\Vert P_1(u^{n-1}_{11},u^{n-1}_{12},u^{n}_{13})-P_1(u^{n-1}_{11},u^{n-1}_{12},u^{n-1}_{13})\Vert \nonumber \\&\quad \le \lambda _{g_1}\Vert x^{n}_1-x^{n-1}_1\Vert +\lambda _{H_1}\lambda _{g_1}\Vert x^{n}_1-x^{n-1}_1\Vert +\lambda _1\lambda _{F_{11}}\Vert x^{n}_1-x^{n-1}_1\Vert +\lambda _1\lambda _{F_{12}}\Vert x^{n}_{2}\nonumber \\&\quad -x^{n-1}_{2}\Vert +\lambda _1\lambda _{F_{13}}\Vert x^{n}_{3}-x^{n-1}_{3}\Vert +\lambda _1\lambda _{P_{11}}\Vert u^{n}_{11}-u^{n-1}_{11}\Vert +\lambda _1\lambda _{P_{12}}\Vert u^{n}_{12}-u^{n-1}_{12}\Vert \nonumber \\&\quad +\lambda _1\lambda _{P_{13}}\Vert u^{n}_{13}-u^{n-1}_{13}\Vert \nonumber \\&\quad \le \lambda _{g_1}\Vert x^{n}_1-x^{n-1}_1\Vert +\lambda _{H_1}\lambda _{g_1}\Vert x^{n}_1-x^{n-1}_1\Vert +\lambda _1\lambda _{F_{11}}\Vert x^{n}_1-x^{n-1}_1\Vert +\lambda _1\lambda _{F_{12}}\Vert x^{n}_2\nonumber \\&\quad -x^{n-1}_2\Vert +\lambda _1\lambda _{F_{13}}\Vert x^{n}_3-x^{n-1}_3\Vert +\lambda _1\lambda _{P_{11}}\left( 1+\frac{1}{n}\right) D_1(A_{11}(x^{n}_{1}),A_{11}(x^{n-1}_{1}))\nonumber \\&\quad +\lambda _1\lambda _{P_{12}}\left( 1+\frac{1}{n}\right) D_2(A_{12}(x^{n}_{2}),A_{12}(x^{n-1}_{2}))\nonumber \\&\quad +\lambda _1\lambda _{P_{13}}\left( 1+\frac{1}{n}\right) D_3(A_{13}(x^{n}_{3}),A_{13}(x^{n-1}_{3}))\nonumber \\&\quad \le \lambda _{g_{1}}\Vert x^{n}_{1}-x^{n-1}_{1}\Vert +\lambda _{H_1}\lambda _{g_{1}}\Vert x^{n}_1-x^{n-1}_1\Vert +\lambda _1\lambda _{F_{11}}\Vert x^{n}_1-x^{n-1}_1\Vert \nonumber \\&\quad +\lambda _1\lambda _{F_{12}}\Vert x^{n}_2-x^{n-1}_2\Vert +\lambda _{1}\lambda _{F_{13}}\Vert x^{n}_{3}-x^{n-1}_{3}\Vert \nonumber \\&\quad +\lambda _1\lambda _{P_{11}}\delta _{A_{11}}\left( 1+\frac{1}{n}\right) \Vert x^{n}_{1}-x^{n-1}_{1}\Vert +\lambda _1\lambda _{P_{12}}\left( 1+\frac{1}{n}\right) \delta _{A_{12}}\Vert x^{n}_{2}-x^{n-1}_{2}\Vert \nonumber \\&\quad +\lambda _{1}\lambda _{P_{13}}\delta _{A_{13}}\left( 1+\frac{1}{n}\right) \Vert x^{n}_{3}-x^{n-1}_{3}\Vert \nonumber \\&\quad \le \left( \lambda _{g_1}+\lambda _1\lambda _{F_{11}}+\lambda _{H_1}\lambda _{g_1}+\lambda _1\lambda _{P_{11}}\delta _{A_{11}}\left( 1+\frac{1}{n}\right) \right) \Vert x^{n}_1-x^{n-1}_1\Vert \nonumber \\&\quad +\left( \lambda _1\lambda _{F_{12}}+\lambda _1\lambda _{P_{12}}\delta _{A_{12}}\left( 1+\frac{1}{n}\right) \right) \Vert x^{n}_2-x^{n-1}_2\Vert \nonumber \\&\quad +\left( \lambda _1\lambda _{F_{13}}+\lambda _1\lambda _{P_{13}}\delta _{A_{13}}\left( 1+\frac{1}{n}\right) \right) \Vert x^{n}_3-x^{n-1}_3\Vert . \end{aligned}$$

Using (17) and (18), (16) becomes

19 $$\begin{aligned} \Vert x^{n+1}_{1}-x^{n}_{1}\Vert &\le {} \left( 1-\mu _1+\mu _1h_1+\mu _1\sqrt{1-2\xi _1+\lambda ^{2}_{g_1}}\right. \nonumber \\&\left. +\frac{\mu _1(\lambda _{g_1}+\lambda _1\lambda _{F_{11}} +\lambda _{H_1}\lambda _{g_1}+\lambda _1\lambda _{P_{11}}\delta _{A_{11}}(1 +\frac{1}{n}))}{1+r_1}\right) \Vert x^{n}_1-x^{n-1}_1\Vert \nonumber \\&+\frac{\mu _1(\lambda _1\lambda _{F_{12}}+\lambda _1\lambda _{P_{12}}\delta _{A_{12}}(1+\frac{1}{n}))}{1+r_1}\Vert x^{n}_2-x^{n-1}_2\Vert \nonumber \\&+\frac{\mu _1(\lambda _1\lambda _{F_{13}}+\lambda _1\lambda _{P_{13}}\delta _{A_{13}}(1+\frac{1}{n}))}{1+r_1}\Vert x^{n}_3-x^{n-1}_3\Vert \nonumber \\&+\mu _1\Vert e^{n}_{1}-e^{n-1}_{1}\Vert . \end{aligned}$$

Using the same arguments as for (19), we have

20 $$\begin{aligned} \Vert x^{n+1}_{2}-x^{n}_{2}\Vert & \le {} \frac{\mu _2(\lambda _2\lambda _{F_{21}}+\lambda _2\lambda _{P_{21}}\delta _{A_{21}}(1+\frac{1}{n}))}{1+r_2}\Vert x^{n}_1-x^{n-1}_1\Vert \nonumber \\&+\left( 1-\mu _2+\mu _2h_2+\mu _2\sqrt{1-2\xi _2+\lambda ^{2}_{g_2}}\right. \nonumber \\&\left. +\frac{\mu _2(\lambda _{g_2}+\lambda _2\lambda _{F_{22}}+\lambda _{H_2}\lambda _{g_2}+\lambda _2\lambda _{P_{22}}\delta _{A_{22}}(1+\frac{1}{n}))}{1+r_2}\right) \Vert x^{n}_2-x^{n-1}_2\Vert \nonumber \\&+\frac{\mu _2(\lambda _2\lambda _{F_{23}}+\lambda _2\lambda _{P_{23}}\delta _{A_{23}}(1+\frac{1}{n}))}{1+r_2}\Vert x^{n}_3-x^{n-1}_3\Vert \nonumber \\&+\mu _2\Vert e^{n}_{2}-e^{n-1}_{2}\Vert . \end{aligned}$$

Using the same arguments as for (19), we have

21 $$\begin{aligned} \Vert x^{n+1}_{3}-x^{n}_{3}\Vert\ &\le {} \frac{\mu _3(\lambda _3\lambda _{F_{31}}+\lambda _3\lambda _{P_{31}}\delta _{A_{31}}(1+\frac{1}{n}))}{1+r_3}\Vert x^{n}_1-x^{n-1}_1\Vert \nonumber \\&+\frac{\mu _3(\lambda _3\lambda _{F_{32}}+\lambda _3\lambda _{P_{32}}\delta _{A_{32}}(1+\frac{1}{n}))}{1+r_3}\Vert x^{n}_2-x^{n-1}_2\Vert \nonumber \\&+\left( 1-\mu _3+\mu _3h_3+\mu _3\sqrt{1-2\xi _3+\lambda ^{2}_{g_3}}\right. \nonumber \\&\left. +\frac{\mu _3(\lambda _{g_3}+\lambda _3\lambda _{F_{33}}+\lambda _{H_3}\lambda _{g_3}+\lambda _3\lambda _{P_{33}}\delta _{A_{33}}(1+\frac{1}{n}))}{1+r_3}\right) \Vert x^{n}_3-x^{n-1}_3\Vert \nonumber \\&+\mu _3\Vert e^{n}_{3}-e^{n-1}_{3}\Vert . \end{aligned}$$

Combining (19) to (21), we have

$$\begin{aligned}&\Vert x^{n+1}_{1}-x^{n}_{1}\Vert +\Vert x^{n+1}_{2}-x^{n}_{2}\Vert +\Vert x^{n+1}_{3}-x^{n}_{3}\Vert \nonumber \\&\quad \le \left( 1-\mu _1+\mu _1h_1+\mu _1\sqrt{1-2\xi _1+\lambda ^{2}_{g_1}}\right. \nonumber \\&\left. \quad +\frac{\mu _1(\lambda _{g_1}+\lambda _1\lambda _{F_{11}}+\lambda _{H_1}\lambda _{g_1}+\lambda _1\lambda _{P_{11}}\delta _{A_{11}}(1+\frac{1}{n}))}{1+r_1}\right) \Vert x^{n}_1-x^{n-1}_1\Vert \nonumber \\&\quad +\frac{\mu _1(\lambda _1\lambda _{F_{12}}+\lambda _1\lambda _{P_{12}}\delta _{A_{12}}(1+\frac{1}{n}))}{1+r_1}\Vert x^{n}_2-x^{n-1}_2\Vert \nonumber \\&\quad +\frac{\mu _1(\lambda _1\lambda _{F_{13}}+\lambda _1\lambda _{P_{13}}\delta _{A_{13}}(1+\frac{1}{n}))}{1+r_1}\Vert x^{n}_3-x^{n-1}_3\Vert \nonumber \\&\quad +\frac{\mu _2(\lambda _2\lambda _{F_{21}}+\lambda _2\lambda _{P_{21}}\delta _{A_{21}}(1+\frac{1}{n}))}{1+r_2}\Vert x^{n}_1-x^{n-1}_1\Vert \nonumber \\&\quad +\left( 1-\mu _2+\mu _2h_2+\mu _2\sqrt{1-2\xi _2+\lambda ^{2}_{g_2}}\right. \nonumber \\&\quad \left. +\frac{\mu _2(\lambda _{g_2}+\lambda _2\lambda _{F_{22}}+\lambda _{H_2}\lambda _{g_2} +\lambda _2\lambda _{P_{22}}\delta _{A_{22}}(1+\frac{1}{n}))}{1+r_2}\right) \Vert x^{n}_2-x^{n-1}_2\Vert \nonumber \\&\quad +\frac{\mu _2(\lambda _2\lambda _{F_{23}}+\lambda _2\lambda _{P_{23}}\delta _{A_{23}}(1+\frac{1}{n}))}{1+r_2}\Vert x^{n}_3-x^{n-1}_3\Vert \nonumber \\&\quad +\frac{\mu _3(\lambda _3\lambda _{F_{31}}+\lambda _3\lambda _{P_{31}}\delta _{A_{31}}(1+\frac{1}{n}))}{1+r_3}\Vert x^{n}_1-x^{n-1}_1\Vert \nonumber \\&\quad +\frac{\mu _3(\lambda _3\lambda _{F_{32}}+\lambda _3\lambda _{P_{32}}\delta _{A_{32}}(1+\frac{1}{n}))}{1+r_3}\Vert x^{n}_2-x^{n-1}_2\Vert \nonumber \\&\quad +\left( 1-\mu _3+\mu _3h_3+\mu _3\sqrt{1-2\xi _3+\lambda ^{2}_{g_3}}\right. \nonumber \\&\quad \left. +\frac{\mu _3(\lambda _{g_3}+\lambda _3\lambda _{F_{33}}+\lambda _{H_3}\lambda _{g_3}+\lambda _3\lambda _{P_{33}}\delta _{A_{33}}(1+\frac{1}{n}))}{1+r_3}\right) \Vert x^{n}_3-x^{n-1}_3\Vert \nonumber \\&\quad +\mu _1\Vert e^{n}_{1}-e^{n-1}_{1}\Vert +\mu _2\Vert e^{n}_{2}-e^{n-1}_{2}\Vert +\mu _3\Vert e^{n}_{3}-e^{n-1}_{3}\Vert \nonumber \\&\quad =\left( 1-\mu _1+\mu _1h_1+\mu _1\sqrt{1-2\xi _1+\lambda ^{2}_{g_1}}+\frac{\mu _1\lambda _{g_1}+\mu _1\lambda _{H_1}\lambda _{g_1}}{1+r_1}+\frac{\mu _1\lambda _1\lambda _{F_{11}}}{1+r_1}\right. \nonumber \\&\quad +\frac{\mu _2\lambda _2\lambda _{F_{21}}}{1+r_2}+\frac{\mu _3\lambda _3\lambda _{F_{31}}}{1+r_3}\nonumber \\&\quad \left. +\left( \frac{\mu _1\lambda _1\lambda _{P_{11}}\delta _{A_{11}}}{1+r_1} +\frac{\mu _2\lambda _2\lambda _{P_{21}}\delta _{A_{21}}}{1+r_2} +\frac{\mu _3\lambda _3\lambda _{P_{31}}\delta _{A_{31}}}{1+r_3}\right) \left( 1 +\frac{1}{n}\right) \right) \Vert x^{n}_1-x^{n-1}_1\Vert \nonumber \\&\quad +\left( 1-\mu _2+\mu _2h_2+\mu _2\sqrt{1-2\xi _2+\lambda ^{2}_{g_2}}+\frac{\mu _2\lambda _{g_2}+\mu _2\lambda _{H_2}\lambda _{g_2}}{1+r_2}\right. \nonumber \\&\quad +\frac{\mu _1\lambda _1\lambda _{F_{12}}}{1+r_1}+\frac{\mu _2\lambda _2\lambda _{F_{22}}}{1+r_2}+\frac{\mu _3\lambda _3\lambda _{F_{32}}}{1+r_3}\nonumber \\&\quad \left. +\left( \frac{\mu _1\lambda _1\lambda _{P_{12}}\delta _{A{12}}}{1+r_1}+\frac{\mu _2\lambda _2\lambda _{P_{22}}\delta _{A_{22}}}{1+r_2}+\frac{\mu _3\lambda _3\lambda _{P_{32}}\delta _{A_{32}}}{1+r_3}\right) \left( 1+\frac{1}{n}\right) \right) \Vert x^{n}_2-x^{n-1}_2\Vert \nonumber \\&\quad +\left( 1-\mu _3+\mu _3h_3+\mu _3\sqrt{1-2\xi _3+\lambda ^{2}_{g_3}}\right. \nonumber \\&\quad +\frac{\mu _3\lambda _{g_3}+\mu _3\lambda _{H_3}\lambda _{g_3}}{1+r_3} +\frac{\mu _1\lambda _1\lambda _{F_{13}}}{1+r_1}+\frac{\mu _2\lambda _2\lambda _{F_{23}}}{1+r_2}+\frac{\mu _3\lambda _3\lambda _{F_{33}}}{1+r_3}\nonumber \\ \end{aligned}$$

$$\begin{aligned}&\quad \left. +\left( \frac{\mu _1\lambda _1\lambda _{P_{13}}\delta _{A_{13}}}{1+r_1} +\frac{\mu _2\lambda _2\lambda _{P_{23}}\delta _{A_{23}}}{1+r_2} +\frac{\mu _3\lambda _3\lambda _{P_{33}}\delta _{A_{33}}}{1+r_3}\right) \left( 1+\frac{1}{n}\right) \right) \Vert x^{n}_3-x^{n-1}_3\Vert \nonumber \\&\quad +\mu _1\Vert e^{n}_{1}-e^{n-1}_{1}\Vert +\mu _2\Vert e^{n}_{2}-e^{n-1}_{2}\Vert +\mu _3\Vert e^{n}_{3}-e^{n-1}_{3}\Vert , \end{aligned}$$

which implies that

22 $$\begin{aligned} \sum \limits ^{3}_{i=1}\Vert x^{n+1}_{i}-x^{n}_{i}\Vert &\le \sum \limits ^{3}_{i=1}\left( 1-\mu _i+\mu _ih_i+\mu _i\sqrt{1-2\xi _i+\lambda ^{2}_{g_i}}+\frac{\mu _i\lambda _{g_i}+\mu _i\lambda _{H_i}\lambda _{g_i}}{1+r_i}\right. \\& \quad + \left. \sum ^{3}_{j=1}\frac{\mu _j\lambda _j\lambda _{F_{ji}}}{1+r_j}+\sum ^{3}_{j=1}\frac{\mu _j\lambda _j\lambda _{P_{ji}}\delta _{A_{ji}}}{1+r_j}\left( 1+\frac{1}{n}\right) \right) \Vert x^{n}_i-x^{n-1}_i\Vert \\& \quad + \sum ^{3}_{i=1}\mu _i\Vert e^{n}_{i}-e^{n-1}_{i}\Vert \\ & \quad \le \sum \limits ^{3}_{i=1}(\kappa _i+\nu ^{n}_{i})\Vert x^{n}_i-x^{n-1}_i\Vert +\sum ^{3}_{i=1}\mu _i\Vert e^{n}_{i}-e^{n-1}_{i}\Vert , \end{aligned}$$

where $\kappa _i=1-\mu _i+\mu _ih_i+\mu _i\sqrt{1-2\xi _i+\lambda ^{2}_{g_i}}+\frac{\mu _i\lambda _{g_i}+\mu _i\lambda _{H_i}\lambda _{g_i}}{1+r_i}+\sum \nolimits ^{3}_{j=1}\frac{\mu _j\lambda _j\lambda _{F_{ji}}}{1+r_j}$ and $\nu ^{n}_{i}=\sum \nolimits ^{3}_{j=1}\frac{\mu _j\lambda _j\lambda _{P_{ji}}\delta _{A_{ji}}}{1+r_j}\left( 1+\frac{1}{n}\right) .$ It follows from (22) that

23 $$\begin{aligned} \sum \limits ^{3}_{i=1}\Vert x^{n+1}_{i}-x^{n}_{i}\Vert \le \sum \limits ^{3}_{i=1}\alpha ^{n}\Vert x^{n}_i-x^{n-1}_i\Vert +\sum ^{3}_{i=1}\mu _i\Vert e^{n}_{i}-e^{n-1}_{i}\Vert , \end{aligned}$$

where

$$\begin{aligned} \alpha ^{n}=\max \{\kappa _1+\nu ^{n}_{1},\kappa _2+\nu ^{n}_{2},\kappa _3+\nu ^{n}_{3}\}, \quad for\,all\, n=1,2,3,\cdots . \end{aligned}$$

Letting $$\alpha =\max \{\kappa _1+\nu _{1},\kappa _2+\nu _{2},\kappa _3+\nu _{3}\},$$ where

$$\begin{aligned} \nu _i=\mu _i\sum ^{3}_{j=1}\frac{\mu _j\lambda _j\lambda _{P_{ji}}\delta _{A_{ji}}}{1+r_j}, \quad for\,each\,\quad i\in \{1,2,3\}, \end{aligned}$$

then $\alpha ^{n}\rightarrow \alpha$ and $\nu ^{n}_{i}\rightarrow \nu _i,$ as $n\rightarrow \infty ,$ for each $i\in \{1,2,3\}.$ From condition (15), we know that $0<\alpha <1$ and hence there exist $n_0\in {\mathbb {N}}$ and $\alpha _0\in (\alpha ,1)$ such that $\alpha ^n\le \alpha _0$ for all $n\ge n_0.$ Therefore, it follows from (23) that

$$\begin{aligned} \sum \limits ^{3}_{i=1}\Vert x^{n+1}_{i}-x^{n}_{i}\Vert &\le {} \sum \limits ^{3}_{i=1}\alpha _{n_0}\Vert x^{n}_i-x^{n-1}_i\Vert +\sum ^{3}_{i=1}\mu _i\Vert e^{n}_{i}-e^{n-1}_{i}\Vert ,\quad for\,all\, n\ge n_0, \end{aligned}$$

which implies that

$$\begin{aligned} \sum \limits ^{3}_{i=1}\Vert x^{n+1}_{i}-x^{n}_{i}\Vert \le \sum \limits ^{3}_{i=1}\alpha ^{n-n_0}_{0}\Vert x^{n_0+1}_i-x^{n_0}_i\Vert +\sum ^{n-n_0}_{p=1}\sum ^{3}_{i=1}\mu _i\alpha ^{p-1}_{0}\iota ^{n-(p-1)}_{i},\quad for\,all\, n\ge n_0, \end{aligned}$$

where $\iota ^{n}_{i}=\Vert e^{n}_{i}-e^{n-1}_{i}\Vert ,$ for all $n\ge n_0.$ Hence, for any $m\ge n>n_0,$ we have

24 $$\begin{aligned} \sum \limits ^{3}_{i=1}\Vert x^{m}_{i}-x^{n}_{i}\Vert &\le {} \sum \limits ^{m-1}_{q=n}\sum \limits ^{3}_{i=1}\Vert x^{q+1}_{i}-x^{q}_{i}\Vert \nonumber \\ &\le {} \sum \limits ^{m-1}_{q=n}\sum \limits ^{3}_{i=1}\alpha ^{q-n_0}_{0}\Vert x^{n_0+1}_{i}-x^{n_0}_{i}\Vert +\sum \limits ^{m}_{q=n}\sum ^{q-n_0}_{p=1}\sum ^{3}_{i=1}\mu _i\alpha ^{p-1}_{0}\iota ^{q-(p-1)}_{i}\nonumber \\ &\le {} \sum \limits ^{m-1}_{q=n}\sum \limits ^{3}_{i=1}\alpha ^{q-n_0}_{0}\Vert x^{n_0+1}_{i}-x^{n_0}_{i}\Vert \nonumber \\+ & {} \sum \limits ^{m}_{q=n}\sum ^{q-n_0}_{p=1}\sum ^{3}_{i=1}\mu _i\alpha ^{q}_{0}\frac{\iota ^{q-(p-1)}_{i}}{\alpha ^{q-(p-1)}_{0}}. \end{aligned}$$

Since $$\sum \nolimits ^{\infty }_{q=1}\iota ^{q}_{1}\kappa ^{-q}<\infty ,$$$$\sum \nolimits ^{\infty }_{q=1}\iota ^{q}_{2}\kappa ^{-q}<\infty ,$$ and $$\sum \nolimits ^{\infty }_{q=1}\iota ^{q}_{3}\kappa ^{-q}<\infty ,$$ for all $$\kappa \in (0,1),$$ and $$\alpha _0<1,$$ it follows from (24) that $$\Vert x^{m}_{1}-x^{n}_{1}\Vert \rightarrow 0,$$$$\Vert x^{m}_{2}-x^{n}_{2}\Vert \rightarrow 0$$ and $$\Vert x^{m}_{3}-x^{n}_{3}\Vert \rightarrow 0,$$ as $$n\rightarrow \infty ,$$ and so $$\{x^{n}_{1}\},$$$$\{x^{n}_{2}\}$$ and $$\{x^{n}_{3}\}$$ are Cauchy sequences in $$X_1,$$$$X_2$$ and $$X_3,$$ respectively. Thus, there exist $$x_1\in X_1,$$$$x_2\in X_2$$ and $$x_3\in X_3$$ such that $$x^{n}_{1}\rightarrow x_1,$$$$x^{n}_{2}\rightarrow x_2$$ and $$x^{n}_{3}\rightarrow x_3,$$ as $$n\rightarrow \infty .$$

Now, we prove that $$u^{n}_{i1}\rightarrow u_{i1}\in A_{i1}(x_1),$$$$u^{n}_{i2}\rightarrow u_{i2}\in A_{i2}(x_2),$$$$u^{n}_{i3}\rightarrow u_{i3}\in A_{i3}(x_3),$$ for each $$i\in \{1,2,3\}.$$ In fact, it follows from the Lipschitz continuity of $$A_{i1},$$$$A_{i2},$$$$A_{i3}$$ and (11)–(13) that

25 $$\begin{aligned} \Vert u^{n}_{i1}-u^{n-1}_{i1}\Vert &\le {} \left( 1+\frac{1}{n+1}\right) \delta _{A_{i1}}\Vert x^{n}_1-x^{n-1}_{1}\Vert , \end{aligned}$$

26 $$\begin{aligned} \Vert u^{n}_{i2}-u^{n-1}_{i2}\Vert &\le {} \left( 1+\frac{1}{n+1}\right) \delta _{A_{i2}}\Vert x^{n}_2-x^{n-1}_{2}\Vert , \end{aligned}$$

27 $$\begin{aligned} \Vert u^{n}_{i3}-u^{n-1}_{i3}\Vert &\le {} \left( 1+\frac{1}{n+1}\right) \delta _{A_{i3}}\Vert x^{n}_3-x^{n-1}_{3}\Vert ,\quad for\,each\,i\in \{1,2,3\}. \end{aligned}$$

From (25)–(27), we know that $$\{u^{n}_{i1}\},$$$$\{u^{n}_{i2}\}$$ and $$\{u^{n}_{i3}\}$$ are also Cauchy sequences. Therefore, there exist $$u_{i1}\in X_1,$$$$u_{i2}\in X_2$$ and $$u_{i3}\in X_3$$ such that $$u^{n}_{i1}\rightarrow u_{i1},$$$$u^{n}_{i2}\rightarrow u_{i2},$$$$u^{n}_{i3}\rightarrow u_{i3},$$ as $$n\rightarrow \infty .$$

Further, for each $i\in \{1,2,3\},$

$$\begin{aligned} d(u_{i1},A_{i1}(x_1)) & \le {} \Vert u_{i1}-u^{n}_{i1}\Vert +d(u^{n}_{i1},A_{i1}(x_1))\\ & \le {} \Vert u_{i1}-u^{n}_{i1}\Vert +D_1(A_{i1}(x^{n}_{1}),A_{i1}(x_1))\\& \le {} \Vert u_{i1}-u^{n}_{i1}\Vert +\left( 1+\frac{1}{n+1}\right) \delta _{A_{i1}}\Vert x^{n}_1-x_{1}\Vert \rightarrow 0,\,as\,n\rightarrow \infty . \end{aligned}$$

Since $$A_{i1}$$ is closed, we have $$u_{i1}\in A_{i1}(x_1).$$ Similarly, $$u_{i2}\in A_{i2}(x_2),$$$$u_{i3}\in A_{i3}(x_3),$$ respectively. By continuity of the mappings $$g_i,$$$$H_i,$$$$F_i,$$$$P_i,$$$$R^{I_i-H_i}_{\lambda _i,M_i}$$ and iterative Algorithm 1, we know that $$u_{i1},$$$$u_{i2},$$$$u_{i3}$$ satisfy the following relation:

$$\begin{aligned} g_i(x_i) &= {} R^{I_i-H_i}_{\lambda _i,M_i(.,x_i)}[(I_i-H_i)(g_i(x_i))-\lambda _iF_i(x_1,x_2,x_3)-\lambda _iP_i(u_{i1},u_{i2},u_{i3})]. \end{aligned}$$

By Lemma 1, $(x_1,x_2,x_3,u_{11},u_{12},u_{13},u_{21},u_{22},u_{23},u_{31},u_{32},u_{33})$ is a solution of SGIVI (7). This completes the proof. $\square$

### *Remark 1*

It is to be noted that the techniques used to prove the convergence result Theorem 3 is different than others. For more details, we refer to Shang and Bouffanais (2014a, b).

The following example ensures that all the conditions of Theorem 3 are fulfilled.

### *Example 1*

For each $i\in \{1,2,3\},$ let $X_i={\mathbb {R}}$ and $g_i:X_i\rightarrow X_i$ be the mappings defined by

$$\begin{aligned} g_i(x)=\frac{x}{103i},\quad \forall \,x\in X_i. \end{aligned}$$

Suppose that the mappings $H_i:X_i\rightarrow X_i$ are defined by

$$\begin{aligned} H_i(x)=-\frac{(1+i)x}{2}, \quad \forall \,x\in X_i, \end{aligned}$$

and the mappings $M_i:X_i\times X_i\rightarrow 2^{X_i}$ are defined by

$$\begin{aligned} M_i(x,y)=\frac{(1+i)x}{2},\quad \forall \,(x,y)\in X_i\times X_i. \end{aligned}$$

Then, it is easy to check that $g_i's$ are $\frac{1}{100i}$-Lipschitz continuous and $\frac{1}{105i}$-strongly monotone, $H_i$’s are *i*-Lipschitz continuous and *i*-relaxed Lipschitz continuous, and $M_i$’s are monotone mappings.

In addition, it is easy to verify that for $$\lambda _i=1,$$$$[(I_i-H_i)+M_i(.,y)](X_i)=X_i,$$ which shows that $$M_i$$’s are $$(I_i-H_i)$$-monotone mappings. Hence, the relaxed resolvent operators $$R^{I_i-H_i}_{\lambda _i,M_i}:X_i\rightarrow X_i$$ associated with $$I_i$$, $$H_i$$ and $$M_i$$ are of the form:

$$\begin{aligned} R^{I_i-H_i}_{\lambda _i,M_i}(x)=\frac{x}{2+i},\quad \forall x\in X_i. \end{aligned}$$

It is easy to see that the relaxed resolvent operators defined above are single-valued.

Now,

$$\begin{aligned} \left\| R^{I_i-H_i}_{\lambda _i,M_i}(x)-R^{I_i-H_i}_{\lambda _i,M_i}(y)\right\| & = \left\| \frac{x}{2+i}-\frac{y}{2+i}\right\| \\&= \frac{1}{2+i}\Vert x-y\Vert \\&\le \frac{1}{1+i}\Vert x-y\Vert . \end{aligned}$$

Hence, the resolvent operators $R^{I_i-H_i}_{\lambda _i,M_i}$ are $\frac{1}{1+i}$-Lipschitz continuous.

Let the mappings $F_i:{\mathbb {R}}\times {\mathbb {R}}\times {\mathbb {R}}\rightarrow {\mathbb {R}}$ be defined by

$$\begin{aligned} F_i(x)=\frac{x_1+x_2+x_3+1}{3480i},\quad \forall x=(x_1,x_2,x_3)\in {\mathbb {R}}\times {\mathbb {R}}\times {\mathbb {R}}, \end{aligned}$$

and the mappings $P_i:{\mathbb {R}}\times {\mathbb {R}}\times {\mathbb {R}}\rightarrow {\mathbb {R}}$ be defined by

$$\begin{aligned} P_i(x)=\frac{x_1+x_2+x_3+i+1}{3370i},\quad \forall x=(x_1,x_2,x_3)\in {\mathbb {R}}\times {\mathbb {R}}\times {\mathbb {R}}. \end{aligned}$$

It can be verified that $F_i$’s are $\frac{1}{1150i}$-Lipschitz continuous in first argument, $\frac{1}{2300i}$-Lipschitz continuous in second argument and $\frac{1}{3450i}$-Lipschitz continuous in third argument, $P_i$’s are $\frac{1}{1100i}$-Lipschitz continuous in first argument, $\frac{1}{2200i}$-Lipschitz continuous in second argument and $\frac{1}{3300i}$-Lipschitz continuous in third argument. Suppose that $A_{i1},A_{i2},A_{i3}:{\mathbb {R}}\rightarrow {\mathbb {R}}$ be the identity mappings. Then, clearly $A_{i1}$’s, $A_{i2}$’s and $A_{i3}$’s are 1-$D_i$-Lipschitz continuous mappings. Hence, all the conditions of Theorem 3 are satisfied.

### *Remark 2*

We choose $$\lambda _{g_i}=\frac{1}{100i},$$$$\xi _i=\frac{1}{105i},$$$$\lambda _{H_i}=i,$$$$r_i=i,$$$$\lambda _{F_{i1}}=\frac{1}{1150i},$$$$\lambda _{F_{i2}}=\frac{1}{2300i},$$$$\lambda _{F_{i3}}=\frac{1}{3450i},$$$$\lambda _{P_{i1}}=\frac{1}{1100i},$$$$\lambda _{P_{i2}}=\frac{1}{2200i},$$$$\lambda _{P_{i3}}=\frac{1}{3300i},$$$$\delta _{A_{i1}}=1,$$$$\delta _{A_{i2}}=1,$$$$\delta _{A_{i3}}=1,$$$$\lambda _i=1,$$ one can easily verify that for $$h_i=\frac{1}{1000i}$$ and $$\mu _i=1,$$ the condition (15) of Theorem 3 is satisfied.

### *Remark 3*

We remark that our results can be further considered in Banach spaces and also the techniques of this paper may be helpful for solving a system of *n*-variational inclusions.

## Conclusion

System of variational inclusions can be viewed as natural and innovative generalizations of the system of variational inequalities. Two of the most difficult and important problems related to inclusions are the establishment of generalized inclusions and the development of an iterative algorithm. In this article, a new system of three variational inclusions is introduced and studied which is more general than many existing system of variational inclusions in the literature. An iterative algorithm is established with error terms to approximate the solution of our system, and convergence criteria is also discussed.

We remark that our results are new and useful for further research and one can extend these results in higher dimensional spaces. Much more work is needed in all these areas to develop a sound basis for applications of the system of general variational inclusions in engineering and physical sciences.
